# F. Mason Sones Jr.: The Serendipitous Discovery of Coronary Angiography and Its Lasting Impact on Cardiology

**DOI:** 10.7759/cureus.61080

**Published:** 2024-05-25

**Authors:** Moiud Mohyeldin, Feras O Mohamed, Ahmed Mustafa, Sai Allu, Shitij Shrivastava

**Affiliations:** 1 Internal Medicine, BronxCare Health System, Bronx, USA; 2 Diagnostic and Interventional Radiology, Memorial Hermann-Texas Medical Center (TMC), Houston, USA; 3 Cardiology, Salaam Clinic, Cleveland, USA; 4 Cardiology, Mercy University Hospital, Cork, IRL

**Keywords:** selective coronary arteriography, cardiovascular pioneers, cardiology history, cleveland clinic, coronary artery bypass surgery, interventional cardiology, cardiac catheterization, coronary angiography, f. mason sones jr, historical vignette

## Abstract

F. Mason Sones Jr. (1918-1985) was a pioneering cardiologist whose groundbreaking work revolutionized the field of cardiology. His accidental discovery of coronary angiography in 1958 at the Cleveland Clinic provided physicians with the first clear visualization of coronary arteries in living patients, paving the way for the development of coronary artery bypass surgery and interventional cardiology. This review article explores F. Mason Sones Jr.'s life and career, and his lasting impact on the field of cardiology.

Born in Noxapater, MS, in 1918, F. Mason Sones Jr. attended Western Maryland College (Westminster, MD) and the University of Maryland School of Medicine (Baltimore, MD) before completing his internship and residency at the University Hospital (Baltimore, MD) and Henry Ford Hospital (Detroit, MI), respectively. After serving in the U.S. Army Air Corps during World War II, F. Mason Sones Jr. joined the Cleveland Clinic (Cleveland, OH), in 1950, as the head of pediatric cardiology, where he combined his expertise in cardiac catheterization with his interest in congenital heart disease. F. Mason Sones Jr.'s serendipitous discovery of coronary angiography occurred during a routine cardiac catheterization procedure when he inadvertently injected contrast dye directly into the right coronary artery. Realizing that smaller amounts of dye could safely opacify the coronary arteries, F. Mason Sones Jr. refined and standardized the technique of selective coronary angiography, collaborating with engineers to improve X-ray imaging and establishing protocols that remain the standard of care today. F. Mason Sones Jr.'s work provided the foundation for the development of coronary artery bypass surgery by Dr. René Favaloro and the birth of interventional cardiology, as pioneered by Dr. Andreas Gruentzig. As the director of cardiovascular disease at the Cleveland Clinic (1966-1975), F. Mason Sones Jr. mentored and inspired a generation of cardiologists, cementing his legacy as a visionary leader in the field. Throughout his career, F. Mason Sones Jr. received numerous awards and honors, including the American Medical Association's Scientific Achievement Award and the Gairdner Foundation International Award. He co-founded and served as the first president of the Society for Cardiac Angiography (now SCAI), an organization dedicated to advancing the field of interventional cardiology. This review article pays tribute to F. Mason Sones Jr.'s enduring contributions to the field of cardiology, highlighting his role as a pioneer, innovator, and mentor. His legacy continues to inspire and guide generations of cardiologists in their pursuit of improving patient care and pushing the boundaries of cardiovascular medicine.

## Introduction and background

**Figure 1:**
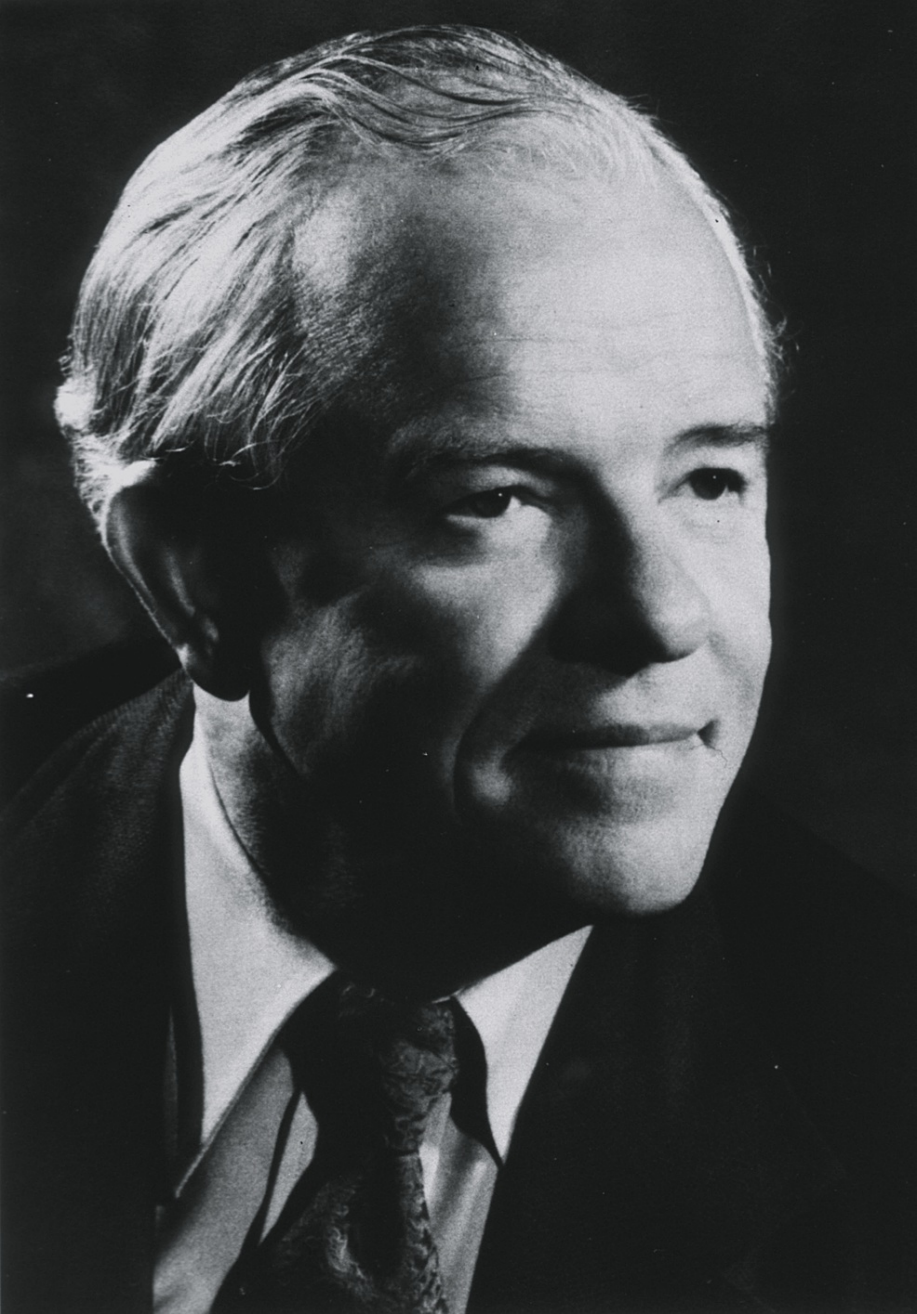
Portrait of F. Mason Sones Jr. F. Mason Sones Jr. was a pioneering cardiologist who revolutionized the field of cardiology with his accidental discovery of coronary angiography in 1958. The image is obtained from the National Library of Medicine (public domain).

Cardiology has witnessed remarkable advancements over the past century, with groundbreaking discoveries and innovations transforming the diagnosis and treatment of cardiovascular diseases. At the forefront of this progress have been visionary physician-scientists whose tireless efforts and ingenuity have pushed the boundaries of medical knowledge and saved countless lives. One such luminary was F. Mason Sones Jr., a pioneering cardiologist whose serendipitous discovery of coronary angiography in 1958 revolutionized the understanding and management of coronary artery disease [1]. Figure 1 shows a portrait of F. Mason Sones Jr., the pioneering cardiologist whose groundbreaking work is the focus of this article.

Prior to F. Mason Sones Jr.'s groundbreaking work, diagnosing and treating coronary artery disease posed significant challenges. Existing diagnostic methods were limited, and treatment options were few, resulting in a dire prognosis for many patients [2]. F. Mason Sones Jr.'s accidental discovery of coronary angiography provided the first clear visualization of coronary arteries in living patients, enabling accurate diagnosis and targeted treatment. Today, over 3 million coronary angiography procedures are performed annually worldwide, a testament to the enduring impact of his work [3,4].

Born in 1918 in Noxapater, MS, F. Mason Sones Jr.'s early life gave little indication of the profound impact he would have on cardiology. After completing his medical degree at the University of Maryland School of Medicine (Baltimore, MD) in 1943, he served in the U.S. Army Air Corps during World War II before beginning his career at the Cleveland Clinic (Cleveland, OH) in 1950 [5]. It was there, as the head of pediatric cardiology, that F. Mason Sones Jr. combined his expertise in cardiac catheterization with a keen eye for innovation to develop a technique that would change the face of cardiology.

F. Mason Sones Jr.'s groundbreaking work in coronary angiography was a fortuitous discovery, born out of a complication during a routine cardiac catheterization procedure. His willingness to challenge conventional wisdom and embrace the unexpected led to a revolution in the understanding and treatment of coronary artery disease. Over the next decade, F. Mason Sones Jr. refined and standardized the technique, collaborating with engineers to improve imaging and establishing protocols that remain the standard of care [6].

F. Mason Sones Jr.'s discovery laid the foundation for the development of coronary artery bypass surgery by Dr. René Favaloro and the birth of interventional cardiology, as pioneered by Dr. Andreas Gruentzig [7,8]. His interdisciplinary approach and commitment to mentoring the next generation of cardiologists cemented his legacy as a visionary leader in the field.

In this article, we will delve into the life and career of F. Mason Sones Jr., exploring his pioneering work in coronary angiography, his collaborations with key figures in the development of coronary artery bypass surgery and interventional cardiology, and his lasting impact on the field of cardiology. Through his story, we will gain insight into the process of medical innovation and the profound influence that a single physician-scientist can have on the course of medical history.

## Review

Early life and education

F. Mason Sones Jr. was born on June 21, 1918, in the small town of Noxapater, MS [8]. Growing up in a rural setting, F. Mason Sones Jr. developed a strong work ethic and a curiosity about the world around him. He attended Western Maryland College (now McDaniel College) in Westminster, MD, where he earned his undergraduate degree in 1940 [9]. F. Mason Sones Jr. then went on to pursue his medical education at the University of Mayland School of Medicine in Baltimore, graduating with his medical degree in 1943 [2].

Early career and military service

After completing his medical degree, F. Mason Sones Jr. began his internship at the University Hospital in Baltimore, where he gained valuable experience in patient care and medical procedures [2]. He then moved to Detroit, MI, for his residency at Henry Ford Hospital (Detroit, MI), where he would later learn the techniques of cardiac catheterization that would prove instrumental in his groundbreaking work [10].

F. Mason Sones Jr.'s early career was interrupted by World War II, during which he served in the U.S. Army Air Corps from 1944 to 1946 [2]. His time in the military provided him with valuable leadership experience and a sense of discipline that would serve him well throughout his career.

Pioneering work in cardiac catheterization at the Cleveland Clinic

In 1950, F. Mason Sones Jr. joined the Cleveland Clinic as the head of pediatric cardiology, a position that would allow him to combine his expertise in cardiac catheterization with his interest in congenital heart disease [11]. At the time, cardiac catheterization was a relatively new technique, and F. Mason Sones Jr. had learned the procedure during his residency at Henry Ford Hospital [10].

F. Mason Sones Jr. quickly established himself as a leader in the field, combining cardiac catheterization with selective arteriography to gain a more comprehensive understanding of the heart's structure and function [12]. His innovative approach laid the foundation for his later work in coronary angiography and cemented his reputation as a pioneer in the field of cardiology.

Accidental discovery of coronary angiography in 1958

**Figure 2:**
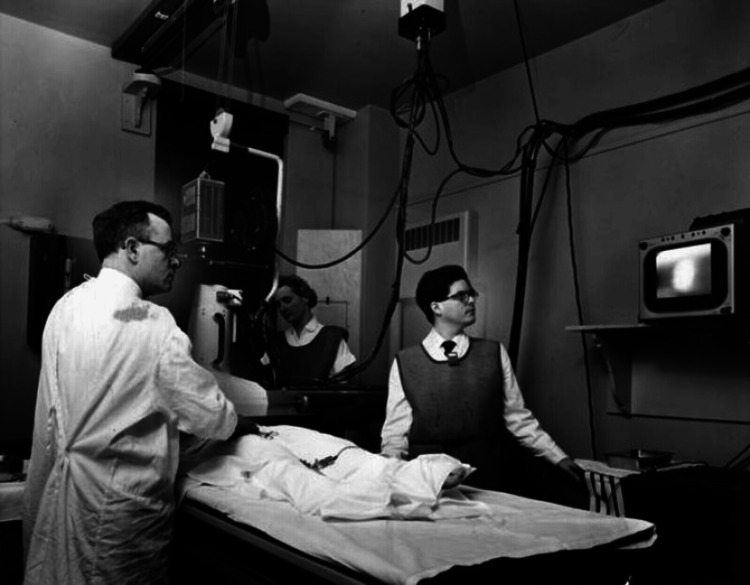
Pioneering coronary angiogram at the Cleveland Clinic. F. Mason Sones Jr. (left) performs an early coronary angiogram at the Cleveland Clinic. The image courtesy of Special Collections, Cleveland State University Library (public domain).

F. Mason Sones Jr.'s most significant contribution to cardiology came in 1958, when he accidentally discovered the technique of coronary angiography during a routine cardiac catheterization procedure [3]. While injecting contrast dye into the aorta of a patient, F. Mason Sones Jr. inadvertently injected the dye directly into the right coronary artery. To his surprise, the patient suffered no ill effects, and the resulting images provided the first clear visualization of the coronary arteries in a living patient [13]. Figure 2 shows F. Mason Sones Jr. (left) performing an early coronary angiogram at the Cleveland Clinic.

This fortuitous event led F. Mason Sones Jr. to realize that smaller amounts of dye could be used to safely opacify the coronary arteries, providing a detailed view of any blockages or abnormalities [14]. Prior to F. Mason Sones Jr.'s discovery, diagnosing and treating coronary artery disease posed significant challenges. Existing diagnostic methods were limited, and treatment options were few, resulting in a dire prognosis for many patients [2]. F. Mason Sones Jr.'s technique provided the first clear images of coronary anatomy, enabling the diagnosis of coronary artery disease and subsequent monitoring of its progression. This laid the foundation for coronary artery bypass grafting and percutaneous coronary interventions, revolutionizing the treatment of coronary artery disease. Today, over three million coronary angiography procedures are performed annually worldwide [3,4].

Refinement and standardization of coronary angiography techniques

Following his accidental discovery, F. Mason Sones Jr. worked tirelessly to refine and standardize the technique of selective coronary angiography. He collaborated with engineers to enhance x-ray imaging, notably through the development of the C-arm. This device significantly improves the visualization of coronary arteries by offering a broader range of imaging angles [15]. The C-arm, a C-shaped apparatus, integrates an X-ray source and detector, allowing for the acquisition of images from multiple angles around the patient. This technological advancement has markedly improved the quality and diagnostic capabilities of coronary angiography [16,17]. 

F. Mason Sones Jr. also established standards of care for selective cine coronary arteriography, ensuring that the procedure was performed safely and effectively [4]. Key elements of these protocols included careful patient selection, meticulous catheterization technique, close monitoring during the procedure, and appropriate post-procedural care. F. Mason Sones Jr.'s protocols became the gold standard for coronary angiography and remain in use to this day.

Impact on the development of coronary artery bypass surgery

F. Mason Sones Jr.'s work in coronary angiography provided the foundation for the development of coronary artery bypass surgery. In 1967, Dr. René Favaloro, a colleague of F. Mason Sones Jr. at the Cleveland Clinic, performed the first successful coronary artery bypass surgery using a saphenous vein graft [18]. This groundbreaking procedure, which relied heavily on the detailed visualization of the coronary arteries provided by F. Mason Sones Jr.'s angiography technique, marked a major milestone in the treatment of coronary artery disease.

F. Mason Sones Jr.'s contributions to the field of cardiology extended beyond his own groundbreaking research. His work provided the foundation for surgical revascularization and inspired a generation of cardiac surgeons to push the boundaries of what was possible in the treatment of heart disease.

Role in the birth of interventional cardiology

In addition to its impact on cardiac surgery, F. Mason Sones Jr.'s discovery of coronary angiography played a crucial role in the birth of interventional cardiology. The ability to visualize the coronary anatomy in detail was essential for the development of percutaneous coronary interventions, such as angioplasty and stenting.

Dr. Andreas Gruentzig, a German cardiologist, built upon F. Mason Sones Jr.'s work when he performed the first successful coronary angioplasty in 1977 [19]. This minimally invasive procedure, which involved inflating a small balloon to widen a narrowed coronary artery, marked the beginning of a new era in the treatment of coronary artery disease.

F. Mason Sones Jr.'s contributions paved the way for the development of a wide range of interventional cardiology techniques, including the use of stents, atherectomy devices, and intravascular imaging. These advances have transformed the field of cardiology and improved the lives of countless patients worldwide.

Collaborative approach and mentorship

Throughout his career, F. Mason Sones Jr. exemplified the importance of collaboration and interdisciplinary teamwork in advancing medical knowledge. His partnerships with engineers were instrumental in refining coronary angiography techniques and improving imaging capabilities [15]. F. Mason Sones Jr. also fostered a culture of collaboration among his colleagues at the Cleveland Clinic, encouraging the exchange of ideas and the pursuit of innovative solutions to complex medical problems.

As the director of cardiovascular disease at the Cleveland Clinic (1966-1975), F. Mason Sones Jr. mentored and inspired a generation of cardiologists [7]. He was known for his dedication to teaching and his commitment to nurturing the next generation of cardiovascular specialists. Many of his trainees went on to become leaders in the field, carrying forward his legacy of innovation and patient-centered care.

According to William Proudfit, a former trainee of F. Mason Sones Jr., Sones was an influential and motivating mentor who inspired his colleagues to strive for excellence in their work. Proudfit described F. Mason Sones Jr. as a dynamic figure in cardiology who had a significant impact on those around him through his dedication to high standards and his role as a teacher [11].

F. Mason Sones Jr.'s leadership extended beyond the walls of the Cleveland Clinic. He co-founded and served as the first president of the Society for Cardiac Angiography (now known as the Society for Cardiovascular Angiography and Interventions, or SCAI), an organization dedicated to advancing the field of interventional cardiology [20]. Through his involvement with SCAI, Sones Jr. helped to establish standards of practice and promote research in the rapidly evolving field of interventional cardiology.

Later career, awards, and legacy

Throughout his distinguished career, Sones Jr. received numerous awards and honors in recognition of his groundbreaking contributions to the field of cardiology. These included the American Medical Association's Scientific Achievement Award, the Gairdner Foundation International Award, and the American College of Cardiology's Distinguished Fellowship Award [21].

Despite these accolades, F. Mason Sones Jr. remained humble and dedicated to his work. He continued to innovate and push the boundaries of cardiovascular medicine until his retirement in 1983. Even after stepping down from his formal leadership roles, F. Mason Sones Jr. remained a trusted advisor and mentor to countless cardiologists who sought his guidance and wisdom [7,8].

F. Mason Sones Jr.'s legacy extends far beyond his individual achievements. His commitment to collaboration, innovation, and patient-centered care set a new standard for cardiovascular medicine and inspired generations of physicians to follow in his footsteps [7]. Today, the techniques and principles he pioneered continue to form the foundation of modern cardiology practice, benefiting millions of patients worldwide [3,4]. Table 1 summarizes the key milestones in F. Mason Sones Jr.'s career and his major contributions to the field of cardiology.

**Table 1:** Timeline of F. Mason Sones Jr.'s career and major contributions to the field of cardiology.

Year	Events
1918	Born in Noxapater, MS
1940	Graduated from Western Maryland College
1943	Earned medical degree from the University of Maryland School of Medicine
1944-1946	Served in the U.S. Army Air Corps
1950	Joined Cleveland Clinic as head of pediatric cardiology
1958	Accidentally discovered coronary angiography
1962	Published landmark paper on selective cine coronary arteriography [4]
1966-1975	Served as director of cardiovascular disease at Cleveland Clinic
1967	Enabled the first successful coronary artery bypass surgery by Dr. René Favaloro
1977	Paved the way for the first successful coronary angioplasty by Dr. Andreas Gruentzig
1985	Passed away at the age of 67 years

## Conclusions

F. Mason Sones Jr.'s serendipitous discovery of coronary angiography ushered in a new era in cardiovascular medicine, enabling for the first time the accurate diagnosis and targeted treatment of coronary artery disease in living patients. His tireless work to refine and standardize the technique, coupled with his collaborative approach and commitment to mentorship, cemented his legacy as a true pioneer in the field. F. Mason Sones Jr.'s story exemplifies the profound impact that curiosity, dedication, and collaboration can have in the face of scientific challenges. His willingness to embrace the unexpected and pursue innovative solutions to complex problems serves as an inspiration to current and future generations of physician-researchers.

As we reflect on F. Mason Sones Jr.'s extraordinary contributions, we are reminded of the enduring power of mentorship and the importance of fostering a culture of innovation in medicine. By building upon the foundation laid by F. Mason Sones Jr. and others, we can continue to push the boundaries of cardiovascular care and improve outcomes for patients worldwide. Although cardiovascular disease remains the leading cause of death globally, the advances pioneered by F. Mason Sones Jr. and his successors have transformed the landscape of cardiology. With continued investment in research, collaboration, and mentorship, we can build upon this legacy and work towards a future where cardiovascular disease is no longer a leading threat to human health.
